# Safety and efficacy of lamivudine/dolutegravir vs. bictegravir/emtricitabine/tenofovir alafenamide in antiretroviral-naive adults with HIV-1 infection in Shanghai, China: a single-centre retrospective study

**DOI:** 10.1099/jmm.0.001949

**Published:** 2025-01-07

**Authors:** Junyang Yang, Lin Wang, Xiaoran Zhang, Li Liu, Yinzhong Shen, Tangkai Qi, Zhenyan Wang, Wei Song, Yang Tang, Shuibao Xu, Jianjun Sun, Youming Chen, Yihong Shen, Jun Chen, Renfang Zhang

**Affiliations:** 1 Department of Infection and Immunology, Shanghai Public Health Clinical Center, Fudan University, Shanghai, PR China; 2 Department of Nursing, Shanghai Public Health Clinical Center, Fudan University, Shanghai, PR China; 3 No. 2 High School of East China Normal University, SongJiang, Shanghai, PR China; 4 Department of Community, Shanyang Community Health Service Center, Shanghai, PR China

**Keywords:** antiretroviral therapy, bictegravir, dual therapy, efficacy, HIV-1, safety

## Abstract

**Introduction.** Lamivudine plus dolutegravir (3TC/DTG) and bictegravir/emtricitabine/tenofovir alafenamide (B/F/TAF) regimens are commonly used as first-line treatments for people living with human immunodeficiency virus (HIV) (PLWH) worldwide.

**Gap Statement.** There are limited comparative data on the antiviral activity and safety between these regimens in ART-naive PLWH, particularly in China, where the 3TC/DTG regimen was integrated into first-line therapy in 2021 and gained broader adoption after its inclusion in the National Health Insurance in 2022.

**Aims.** This study aims to provide real-world evidence comparing the 3TC/DTG regimen to the B/F/TAF regimen in ART-naive PLWH in China.

**Methodology.** This retrospective study enrolled PLWH initiating ART with either 3TC/DTG or B/F/TAF in Shanghai from January 2020 to January 2023. Demographic characteristics and clinical information were collected and compared for each patient.

**Results.** A total of 380 eligible, ART-naive PLWH were included, with 190 patients in the 3TC/DTG group and 190 patients in the B/F/TAF group. Following the initiation of ART, most patients (94.1 and 89.3% for 3TC/DTG and B/F/TAF groups, respectively) achieved viral suppression (<50 copies of HIV RNA per millilitre) at week 24. The CD4 cell count significantly increased from a baseline of 301.3±185.8 cells per microlitre to 479.5±229.3 cells per microlitre at week 36 for the 3TC/DTG group and from 289.2±188.8 cells per microlitre at baseline to 487.8±234.2 cells per microlitre at week 36 for the B/F/TAF group. Both groups experienced an increase in blood lipid levels after initiating ART, with higher levels of high-density lipoprotein cholesterol (HDL-C) observed in the 3TC/DTG group compared with the B/F/TAF group. Renal and hepatic function indicators remained stable in both groups.

**Conclusions.** 3TC/DTG demonstrates similar antiviral efficacy to B/F/TAF and does not significantly impact liver and kidney functions. Patients receiving 3TC/DTG showed higher plasma HDL-C levels compared with those on B/F/TAF, which confer long-term clinical benefits in reducing cardiovascular risk.

## Introduction

Human immunodeficiency virus (HIV) poses a significant threat to the human immune system, leading to devastating immune deficiencies that can result in opportunistic infections and cancers. However, the global advancement and widespread utilization of antiretroviral therapy (ART) have revolutionized HIV management, dramatically reducing mortality rates and markedly increasing the life expectancy of people living with HIV (PLWH) [1]. HIV infection has transitioned from an acute lethal condition to a chronic infectious disease [2]. However, the long-term use of ART drugs is often associated with a range of adverse effects, including metabolic complications, cardiovascular issues, renal and liver toxicity, neurological symptoms, gastrointestinal disturbances, decreased bone density and so on [3,5]. These diverse side effects highlight the importance of careful treatment selection and ongoing patient monitoring to optimize therapy and minimize health risks.

Lamivudine plus dolutegravir (3TC/DTG) regimen and bictegravir/emtricitabine/tenofovir alafenamide (B/F/TAF) regimen are widely employed globally due to their robust antiviral efficacy, minimal adverse effects and favourable tolerability [6,8]. Both regimens have garnered the endorsement of numerous esteemed international guidelines as the first-line treatments for PLWH [9,11]. However, it remains unclear which regimen may provide the optimal balance of superior antiviral efficacy while minimizing drug-related side effects. The reduction of one nucleoside reverse transcriptase inhibitor in the 3TC/DTG regimen raises concerns about a potential increase in the risk of treatment failure. Conversely, the use of TAF in the B/F/TAF regimen has been linked to heightened risks of lipid abnormalities and renal function impairment, making the choice of regimen critical.

Given these considerations, understanding the comparative data is crucial, particularly because therapeutic decisions are increasingly made within the context of evolving treatment guidelines. There is currently limited comparative data on the efficacy and safety of 3TC/DTG and B/F/TAF regimens in ART-naive PLWH, especially in Chinese. 3TC/DTG regimen was incorporated into first-line treatment for HIV patients only in 2021 and has recently seen widespread use in China following its inclusion in the National Health Insurance in 2022 [12]. This study aims to provide real-world evidence comparing the 3TC/DTG regimen to the B/F/TAF regimen in ART-naive PLWH in China.

## Methods

### Study population

This retrospective study included individuals diagnosed with HIV who commenced ART with 3TC/DTG at the Shanghai Public Health Clinical Center from January 2020 to January 2023. Concurrently, patients who initiated ART with the B/F/TAF regimen during the same period were enrolled in a 1 : 1 ratio. To ensure a sufficient follow-up period, this study only included patients with a follow-up time exceeding 6 months, as the majority of patients undergo HIV RNA testing at this time after ART.

Inclusion criteria encompassed proof of a positive HIV test, an age exceeding 16 years and no history of ART exposure. Exclusion criteria include the following: individuals with pre-existing viral resistance mutations to any agents comprising the utilized ART regimens; those who switched to alternative regimens after initial therapy individuals with concomitant tumours and patients currently using immunosuppressive medications. The Shanghai Public Health Clinical Center Ethics Committee granted ethical approval to the study protocol (ethics approval number: 2016-S-044-01). Given the retrospective and anonymous design of this study, the Ethics Committee waived the requirement for written informed consent from the subjects.

### Data collection

Comprehensive baseline and subsequent follow-up data were meticulously documented within patient charts and the Hospital Laboratory Retrieval System, ensuring a robust framework for analysis and assessment. Our data collection encompassed a broad spectrum of variables, including patient demographic characteristics such as gender, age and ART regimens. Additionally, a range of disease-relevant metrics was captured, notably CD4+ and CD8+ T cell counts, HIV viral load and various biochemical parameters including serum total cholesterol (TC), triglycerides (TG), low-density lipoprotein cholesterol (LDL-C), high-density lipoprotein cholesterol (HDL-C), serum creatinine (SCr), cystatin C (cys-c), alanine aminotransferase (ALT), aspartate aminotransferase (AST) and alkaline phosphatase (ALP). The estimated glomerular filtration rate (eGFR) was calculated using the formula eGFR=175×Scr −1.234×age-0.179×0.79 (if female) [13]. Atherogenic index of plasma (AIP) was calculated using the formula AIP=log10 [TG (mmol/l)/HDL-C (mmol/l)]. Disease-relevant information was meticulously collated at follow-up intervals to monitor the progression and response to treatment. For most patients, these intervals were at week 4, 8, 12 and every 12 weeks post-ART initiation.

The primary outcome measured was the proportion of participants achieving virological suppression, defined as an HIV-1 RNA level below 50 copies per millilitre by week 24. Secondary outcomes included immunological response as evidenced by changes in CD4 counts and CD4/CD8 ratios, as well as alterations in TC, TG, LDL-C, HDL-C, AIP, Scr, cys-c, ALT and AST levels.

### Data analysis

Data were analysed using IBM SPSS version 19.0 (IBM SPSS, Inc., Armonk, NY, USA), and visual representations were generated with GraphPad Prism 6.0 software (GraphPad Software Inc., San Diego, CA, USA). Continuous variables were presented as mean values with sd, while categorical variables were expressed in terms of numbers and percentages. Chi-squared test or Fisher’s exact test was used for categorical variables and *t*-test or Mann–Whitney test was used for continuous variables. A conventional *P*-value of 0.05 (two-tailed) was set as the threshold for statistical significance.

To ensure data integrity, we chose not to use specialized methods for managing missing data. Instead, we removed instances with missing data during the statistical analysis. Subsequently, we removed the cases with missing data and retained only complete cases for reanalysis in order to conduct a more robust statistical evaluation (File S1, available in the online Supplementary Material).

## Results

### Participant characteristics

Between January 2020 and May 2023, a total of 380 ART-naive HIV-infected individuals participated in the study, with 190 in 3TC/DTG group and 190 in the B/F/TAF group. The baseline demographic and clinical characteristics exhibited a general homogeneity across both groups. B/F/TAF group demonstrated significantly higher levels of HIV RNA (4.46±0.75 vs. 4.75±0.69 log10 copies per millilitre, *P*<0.001, for 3TC/DTG and B/F/TAF groups, respectively) and eGFR (108.8±27.1 vs. 118.8±23.6, *P*<0.001, for 3TC/DTG and B/F/TAF groups, respectively) (Table 1).

**Table 1.** Comparison of baseline demographic and clinical characteristics in two groups

Group	3TC/DTG (*N*=190)	B/F/TAF (*N*=190)	*P*
Age (year, mean±sd)	39.3±14.4	37.7±12.7	0.259
Gender (male, %)	173 (91.1)	180 (94.7)	0.162
HIV-1 RNA (log10 copies per millilitre, mean±sd)	4.46±0.75	4.75±0.69	<0.001
HVL, >500 000 copies per millilitre, *n* (%)	8 (4.4)	22 (11.6)	0.001
CD4+count (cells per microlitre, mean±sd)	301.3±185.8	289.2±188.8	0.538
>200, *n* (%)	124 (69.6)	130 (68.4)	0.797
CD4/CD8 ratio (mean±sd)	0.33±0.23	0.29±0.20	0.102
ALT (U l^−1^, mean±sd)	29.9±24.6	34.2±38.6	0.302
AST (U l^−1^, mean±sd)	25.6±16.1	28.3±27.9	0.359
ALP (U l^−1^, mean±sd)	76.5±27.2	74.8±22.5	0.543
TBIL (mmol l^−1^, mean±sd)	13.2±7.7	13.8±7.0	0.321
sCr (µmol l^−1^, mean±sd)	78.8±36.6	63.9±12.9	0.144
eGFR (ml∙min^−1^∙1.73 m^−2^)	108.8±27.1	118.8±23.6	<0.001
Cys-c (mg l^−1^, mean±sd)	0.84±0.46	075±0.20	0.197
RBP (mg l^−1^, mean±sd)	46.0±13.2	43.8±10.8	0.557
TC (mmol l^−1^, mean±sd)	4.4±1.0	4.3±0.8	0.581
TG (mmol l^−1^, mean±sd)	1.7±1.1	1.7±1.0	0.691
HDL-C (mmol l^−1^, mean±sd)	1.1±0.3	1.0±0.3	0.040
LDL-C (mmol l^−1^, mean±sd)	2.9±0.9	2.8±0.7	0.805
AIP	0.16±0.32	0.20±0.28	0.176

HVL, high viral load; RBP, retinol-binding protein; TBIL, total bilirubin.

### Comparison of virological suppression

Following the initiation of ART, by week 12, the percentage of participants with HIV RNA <50 copies per millilitre was 77.4% (24/31) in the 3TC/DTG group, which was comparable to 76.9% (20/26) in the B/F/TAF group (*P*=0.965). By week 24, the percentage of participants with HIV RNA <50 copies per millilitre had increased to 94.1% (127/135) and 89.3% (117/131) for 3TC/DTG and B/F/TAF groups, respectively (*P*=0.159). Following week 24, the 3TC/DTG group and the B/F/TAF group underwent 127 and 93 measurements of HIV viral load, respectively. In the 3TC/DTG group, only one patient exhibited a viral load exceeding 1000 copies per millilitre due to the interruption of ART. Low-level viraemia events, defined as a viral load ranging from 50 to 999 copies per millilitre after 6 months of initiating ART, were observed in 2.3% of patients in the 3TC/DTG group and 5.3% in the B/F/TAF group (*P*=0.287).

### Comparison of immunological responses

**Fig. 1.**
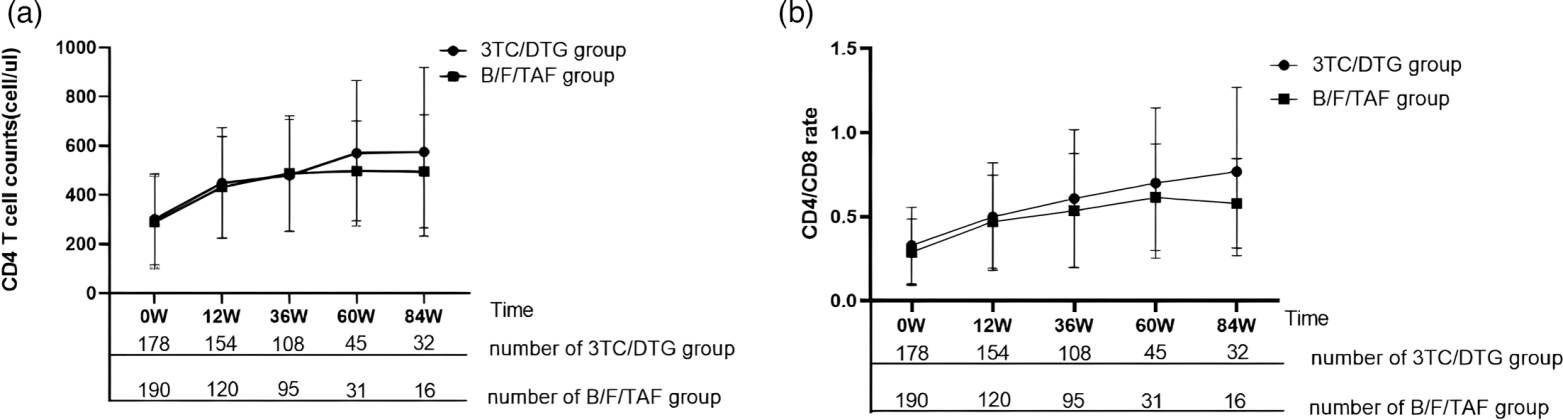
Comparison of immunological response in subgroups. (**a**) Change of CD4+T cell count in 3TC/DTG vs. B/F/TAF group. (**b**) Change of CD4/CD8 ratio in the 3TC/DTG vs. B/F/TAF group.

CD4 cell counts and CD4/CD8 ratios were evaluated at baseline, as well as at weeks 12, 36, 60 and 84. Following ART, a significant increase in CD4 cell counts was observed in both 3TC/DTG group (301.3±185.8 cell ml^−1^ vs. 479.5±229.3 cell ml^−1^, *P*<0.001, for baseline and week 36, respectively) and B/F/TAF group (289.2±188.8 cell ml^−1^ vs. 487.8±234.2 cell ml^−1^, *P*<0.001, for baseline and week 36, respectively). A similar change was also noted in CD4/CD8 ratios in the 3TC/DTG group (0.33±0.23 vs. 0.61±0.41 cell ml^−1^, *P*<0.001, for baseline and week 36, respectively) and the B/F/TAF group (0.29±0.20 vs. 0.54±0.34 cell ml^−1^, *P*<0.001, for baseline and week 36, respectively). No statistically significant differences were found in the changes of CD4 cell counts and CD4/CD8 ratios between the two groups (Fig. 1). We also conducted a reanalysis on patients with complete data at baseline, week 12 and week 36, and similar results were obtained (Fig. S1).

### Serum lipid profile

**Fig. 2.**
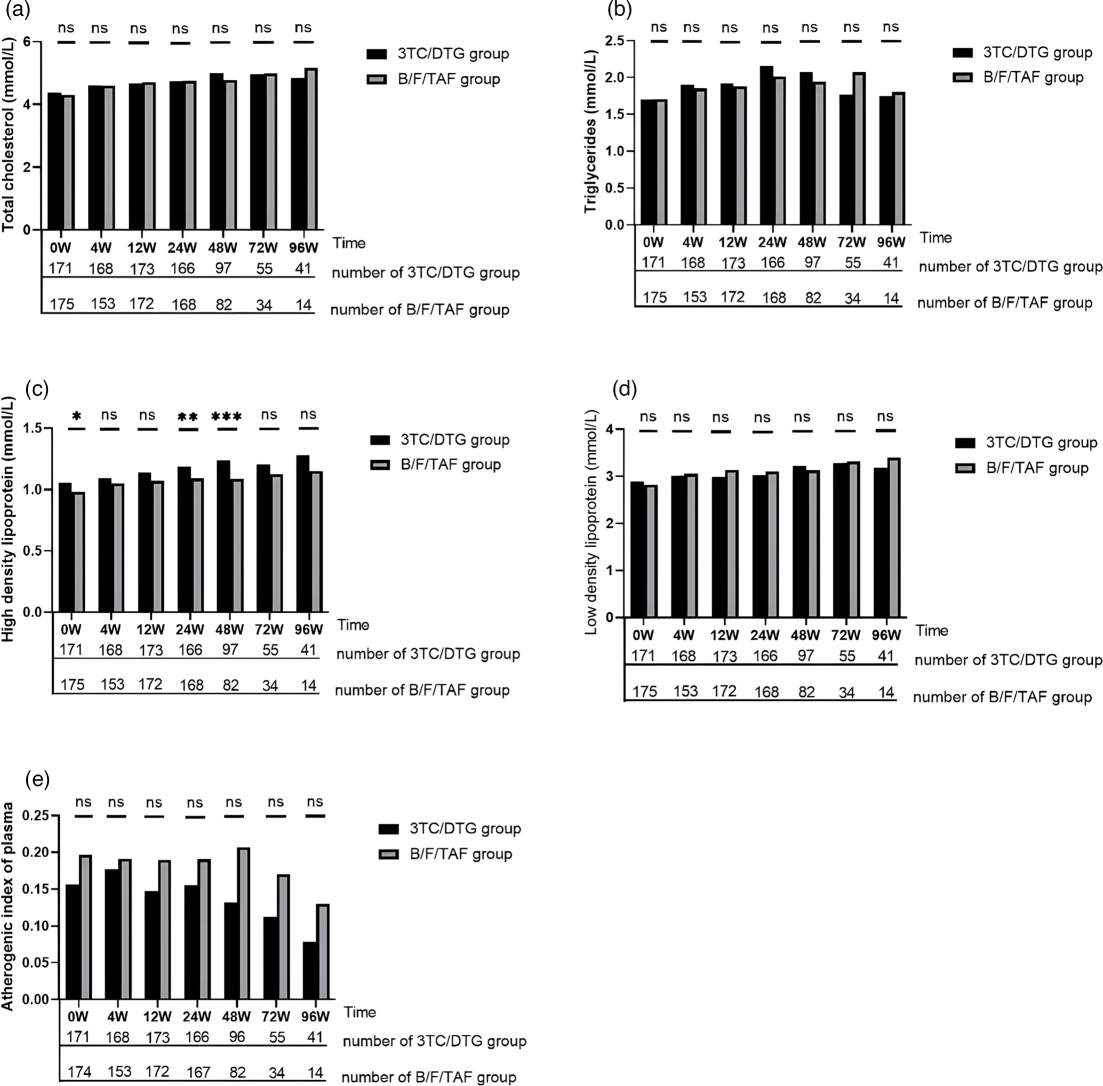
Comparison of lipids in subgroups. (**a**) Change of total cholesterol in 3TC/DTG vs. B/F/TAF group. (**b**) Change of triglyceride in 3TC/DTG vs. B/F/TAF group. (**c**) Change of high-density lipoprotein in 3TC/DTG vs. B/F/TAF group. (**d**) Change of low-density lipoprotein in 3TC/DTG vs. B/F/TAF group. (**e**) Change of atherogenic index of plasma in 3TC/DTG vs. B/F/TAF group. *P<0.05；**P<0.01；***P<0.001.

The levels of TC, TG, LDL-C, HDL-C and AIP were assessed at baseline and weeks 4, 12, 24, 48, 72 and 96 (Fig. 2). A notable increase in TC levels was observed in both 3TC/DTG and B/F/TAF groups from baseline to week 48, with the former group increasing from 4.4±1.0 to 5.0±1.2 mmol l^−1^ (*P*<0.001) and the latter group increasing from 4.3±0.8 to 4.8±0.9 mmol l^−1^ (*P*<0.001). However, there was no significant difference in plasma TC levels between the two groups at week 48 (*P*=0.480).

There were no statistically significant changes in plasma TG levels over the course of 48 weeks in either the 3TC/DTG group (1.7±1.1 to 2.1±2.1 mmol l^−1^, *P*=0.055) or the B/F/TAF group (1.7±1.0 to 1.9±1.0 mmol l^−1^, *P*=0.098). Additionally, plasma TG levels were consistently similar between the groups.

Plasma HDL-C levels demonstrated a statistically significant increase from baseline to week 48 in both groups. Specifically, in the 3TC/DTG group, levels increased from 1.1±0.3 to 1.2±0.3 mmol l^−1^ (*P*<0.001), while in the B/F/TAF group, levels rose from 1.0±0.3 to 1.1±0.3 mmol l^−1^ (*P*=0.005). It is noteworthy that the 3TC/DTG group exhibited slightly higher plasma HDL-C levels at week 48 compared with the B/F/TAF group (*P*=0.005).

Similarly, a significant increase in plasma LDL-C levels was observed from baseline to week 48 in the 3TC/DTG group (2.9±0.9 to 3.2±1.1 mmol l^−1^, *P*=0.006) and the B/F/TAF group (2.8±0.7 to 3.1±0.8 mmol l^−1^, *P*=0.002). However, by week 48, there was no significant difference in plasma LDL-C levels between the two groups (*P*=0.933).

### Renal and hepatic function profiles

**Fig. 3.**
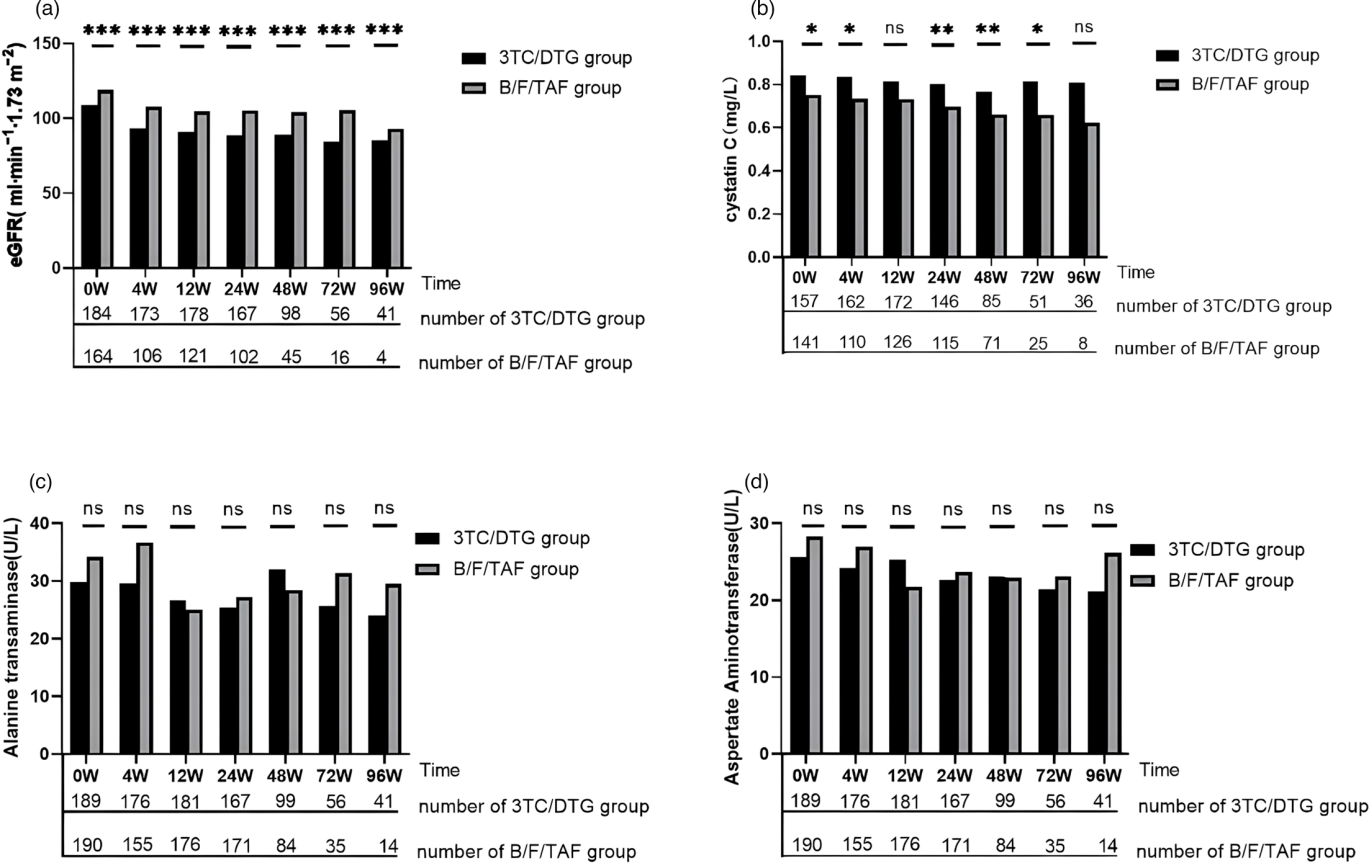
Comparison of renal and hepatic function in subgroups. (**a**) Change of eGFR in 3TC/DTG vs. B/F/TAF group. (**b**) Change of cystatin C in 3TC/DTG vs. B/F/TAF group. (**c**) Change of alanine aminotransferase in 3TC/DTG vs. B/F/TAF group. (**d**) Change of aspartate aminotransferase in 3TC/DTG vs. B/F/TAF group. *P<0.05；**P<0.01；***P<0.001.

Renal and hepatic functions were evaluated at baseline, as well as at weeks 4, 12, 24, 48, 72 and 96 (Fig. 3). A significant decrease in eGFR was observed during the initial 12 weeks following the initiation of ART in both groups. The 3TC/DTG group showed a decrease in eGFR from 108.8±27.1 mmol l^−1^ at baseline to 90.9±20.6 mmol/L at the 12th week (*P*<0.001), while B/F/TAF group exhibited a decrease from 118.8±23.6 mmol l^−1^ to 104.6±14.1 mmol l^−1^ (*P*<0.001). Subsequently, eGFR levels remained relatively stable. Cys-c levels remained stable in the 3TC/DTG group but notably declined from 0.75±0.20 at baseline to 0.66±0.13 at 48th week in the B/F/TAF group. Both ALT and AST levels remain relatively stable after initial ART in both groups.

## Discussion

DTG and Bictegravir (BIC), as forefront contenders in the new generation of integrase inhibitors, bring forth notable advantages with their potent antiviral capabilities, minimal side effects and reduced incidence of drug interactions. These attributes have propelled DTG and BIC to become among the most prevalently utilized ART medications globally. Our study embarked on a comparative evaluation of 3TC/DTG and B/F/TAF regimens among ART-naive PLWH in China, with an anticipation that our findings would enrich the corpus of evidence supporting the long-term management of PLWH. We observed parity in virological suppression and immunological responses between 3TC/DTG and B/F/TAF groups. Moreover, an elevation in HDL-C levels was observed across both regimen groups; however, the increase was more pronounced in the 3TC/DTG group compared to the B/F/TAF group. By the 48-week mark, although TC and LDL-C levels witnessed a significant surge in both groups, the comparative analysis between the two groups revealed no substantial disparity. TG levels and AIP remained consistently analogous across both groups, substantiating the absence of significant differential impact attributable to either treatment regimen. Notably, renal function markers, including eGFR, showed a marked decrease following the initiation of ART, particularly during the first 12 weeks. In contrast, hepatic function indicators, such as ALT and AST, remained largely stable in both groups of patients.

With respect to antiviral efficacy, the contemporary discourse has yet to distil a consensus on whether DTG- or BIC-based regimens reign supreme [14,15]. Our findings indicate that the viral suppression and immunological responses of 3TC/DTG and B/F/TAF regimens are comparable, which aligns with research results from other regions in China [8,16]. Compared to various current 3- or 4-drug ART regimens, 3TC/DTG was shown to be noninferior in maintaining virologic suppression [17]. In the TANGO study, an open-label, multicentre, phase 3 trial, 3TC/DTG regimen demonstrated virologic suppression rates that were not inferior to those of the B/F/TAF regimen [18]. Moreover, we found that the incidence of low-level viraemia in the 3TC/DTG group was lower than that in the B/F/TAF group, although this difference was not statistically significant. This observation is supported by research which indicates that low viraemia may be tied to a viral reservoir that is not fully suppressed by ART, leading to residual immune activation and potential progression of HIV-related complications [19]. In particular, low-level viraemia is associated with adverse clinical outcomes, such as increased risk of virological failure, immune activation and inflammation, which can contribute to non-AIDS defining events and other comorbidities [19,20]. Moreover, intensifying ART did not mitigate the incidence of persistent low-level viraemia in people living with HIV (PWLH) [21,22]. This indicates that lowering the amount of nucleoside reverse transcriptase inhibitor in the 3TC/DTG regimen does not diminish its antiviral effectiveness when compared with the B/F/TAF regimen. Nevertheless, a more extended period of observation is still necessary to confirm its long-term efficacy relative.

In the contemporary medical domain, where a definitive treatment for HIV infection, long-term ART persists as the cornerstone of management for the foreseeable future. Consequently, evaluating the chronic adverse effects associated with ART regimens emerges as a critical metric. In ART-naïve PLWH, RAL and DTG were linked to a smaller rise in TC, LDL-C and TG compared with regimens based on efavirenz and protease inhibitors/ritonavir [23]. Other studies also have shown that B/F/TAF and DTG-based regimens had minimal changes in fasting lipid levels and stable TC/HDL-C ratios, with only a small percentage of participants starting lipid-lowering therapies [24]. In our research, the 3TC/DTG regimen, compared with the B/F/TAF regimen, led to a more significant elevation in plasma HDL-C levels. HDL-C plays a crucial role in promoting the reverse transport of cholesterol, which is essential for clearing cholesterol from the arteries [25]. Elevated HDL-C levels are known to be inversely associated with cardiovascular events and coronary heart disease, suggesting that HDL-C functionality could serve as a better indicator of cardiovascular risk [26]. Furthermore, HDL-C possesses anti-inflammatory and antioxidant properties and improves insulin sensitivity, all of which contribute to the prevention of metabolic and cardiovascular diseases [27]. Considering the extensively documented role of HDL-C in mitigating cardiovascular diseases [28,29], we speculate that 3TC/DTG regimen might reduce the incidence of cardiovascular diseases among HIV patients to some extent. However, a long-term follow-up cohort is needed to confirm our hypothesis.

Renal impairment constitutes a frequent adverse reaction in PLWH commencing ART, predominantly ascribed to the utilization of TDF, known to induce chronic and potentially non-reversible nephropathy [5,30]. 3TC/DTG regimen eschews TDF, thereby diminishing renal burden associated with ART, albeit potentially augmenting hepatitis B risk among HIV cohorts. Contrariwise, the B/F/TAF regimen incorporates TAF, a chemically altered version of TDF, to attenuate nephrotoxicity while sustaining antiviral potency against HIV. In our study, we observed a significant eGFR decline in both groups post-ART initiation. This phenomenon may be attributable to the propensity of both DTG and BIC to impede creatinine’s tubular secretion, thereby precipitating an evident elevation in serological creatinine levels [31]. To more precisely evaluate the impact of ART regimens on renal function, we extended our analysis to the fluctuations of cystatin C levels post-ART initiation [32]. Further analysis revealed that serum cystatin C concentrations remained stable or even decreased in some cases across both groups. This could be indicative of a reduction in peripheral blood HIV viral load due to ART, which may alleviate HIV-associated renal damage and permit partial recovery of kidney function [33]. These findings lend support to the notion that the observed reduction in eGFR is primarily driven by DTG- and BIC-induced suppression of creatinine’s tubular excretion. According to the recommendations of the EACS guidelines, glomerular function can be assessed by using cystatin C-measured eGFR in PLWH on stable ART, which can reduce the impact of ARV drugs or dietary supplements [10].

Our study has several limitations that need to be acknowledged. Primarily, the retrospective nature of our study has led to the omission of pivotal details such as patient weight and drug-associated adverse effects, both of which are instrumental in appraising drug safety profiles. Nevertheless, the extant literature within the same domain suggests that both examined regimens may contribute to weight gain, albeit with a minimal incidence of mild drug-related adverse reactions that typically do not necessitate intervention [8,24, 34]. Our study is constrained by substantial data loss, a brief follow-up period and a relatively small sample size, which could potentially diminish the statistical power of our findings. To verify data reliability, we analysed patients with complete data across all time points, yielding results consistent with the previous findings (Figs S1 and S2). Moreover, the majority of our study participants were adult males, which may limit the generalizability of our findings to females and children. Additionally, our study lacks collected information on patients’ concomitant medications. However, it is important to highlight that both 3TC/DTG and B/F/TAF regimens are associated with minimal drug–drug interaction. Therefore, the lack of detailed data on concomitant medications is not anticipated to significantly affect our findings. Lastly, individuals who switched regimens were excluded, potentially underestimating adverse reactions and overestimating treatment efficacy. These limitations should be carefully considered when interpreting our results. Future research should aim to employ a prospective design, larger sample sizes, extended follow-up periods and a more diverse participant cohort to further validate our findings.

In conclusion, the 3TC/DTG regimen demonstrates comparable antiviral efficacy to the B/F/TAF regimen, achieving similar levels of viral suppression. Low-level viraemia events were slightly lower in the 3TC/DTG group compared with the B/F/TAF group, although this difference was not significant. Furthermore, the 3TC/DTG regimen does not significantly adversely affect liver and kidney function. Patients on the 3TC/DTG regimen had higher plasma HDL-C levels compared with those on the B/F/TAF regimen, while levels of TCH, TG and LD were generally similar across both groups.

## Supplementary material

10.1099/jmm.0.001949 Uncited Supplementary Material 1.

## Abbreviations

AIP: atherogenic index of plasma
ALP: alkaline phosphatase
ALT: alanine aminotransferase
ART: antiretroviral therapy
AST: aspartate aminotransferase
B/F/TAF: bictegravir/emtricitabine/tenofovir alafenamide
BIC: Bictegravir
cys-c: cystatin C
eGFR: estimated glomerular filtration rate
HDL-C: high-density lipoprotein cholesterol
HIV: human immunodeficiency virus
LDL-C: low-density lipoprotein cholesterol
SCr: serum creatinine
TC: total cholesterol
3TC/DTG: lamivudine plus dolutegravir
TG: triglycerides
