# Supplementary material for: Safety and efficacy of lamivudine/dolutegravir vs. bictegravir/emtricitabine/tenofovir alafenamide in antiretroviral-naive adults with HIV-1 infection in Shanghai, China: a single-centre retrospective study
Source: J Med Microbiol. 2025 Jan 7;74(1):001949. doi: 10.1099/jmm.0.001949 (PMC12451750; doi:10.1099/jmm.0.001949)
Supplement: Uncited Supplementary Material 1. [file jmm-74-01949-s001.pdf]

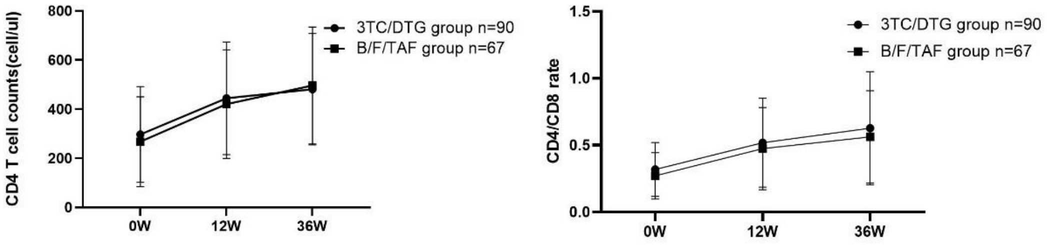

Figure S1. Comparison of immunological response in subgroups for patients with full data at baseline, week 12, and week 36. (A) Change of CD4+ T cell count in 3TC/DTG vs. B/F/TAF group. (B) Change of CD4/CD8 ratio in 3TC/DTG vs. B/F/TAF

group.

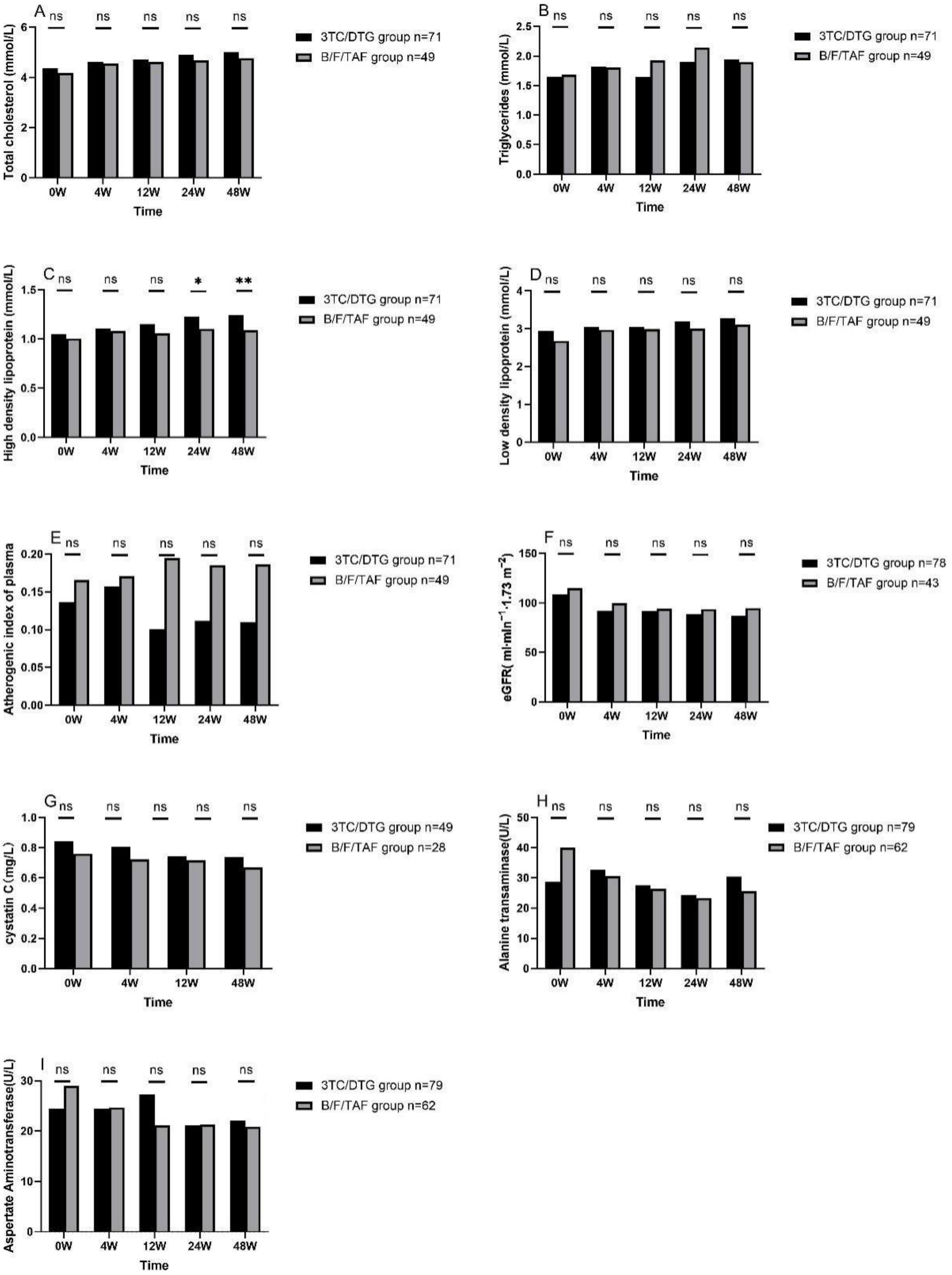

Figure S2. Comparison of lipid, renal function, and hepatic function in subgroups for patients with full data at baseline, week 4, week 12, week 24, and week 36. (A)

Change of total cholesterol in 3TC/DTG vs. B/F/TAF group. (B) Change of triglyceride in 3TC/DTG vs. B/F/TAF group. (C)Change of high-density lipoprotein in 3TC/DTG vs. B/F/TAF group. (D)Change of low-density lipoprotein cholesterol in 3TC/DTG vs. B/F/TAF group. (E)Change of Atherogenic index of plasma in 3TC/DTG vs. B/F/TAF group. (F) Change of eGFRin 3TC/DTG vs. B/F/TAF group. (G) Change of Cystatin C in 3TC/DTG vs. B/F/TAF group. (H) Change of alanine aminotransferase in 3TC/DTG vs. B/F/TAF group. (I) Change of aspartate aminotransferase in 3TC/DTG vs. B/F/TAF group.

*， $P<0.05$； **， $P<0.01$； ***， $P<0.001$
